# Comparative Sequence Analysis of the Envelope Gene of Kyasanur Forest Disease Virus Vaccine Strain With Currently Circulating Field Strains

**DOI:** 10.1155/av/6915402

**Published:** 2026-09-18

**Authors:** Keerthi Kaje, Chandranaik B. Marinaik, Amitha Reena Gomes, Apsana Rizwan, Jagadish Hiremath, Doddamane Rathnamma, Shrikrishna Isloor, Sharanagouda S. Patil, M. Archana

**Affiliations:** ^1^ Institute of Animal Health and Veterinary Biologicals, KVAFSU, Bangalore 560024, India, kvafsu.kar.nic.in; ^2^ Veterinary College, KVAFSU, Bangalore 560024, Karnataka, India, kvafsu.kar.nic.in; ^3^ ICAR-National Institute for Veterinary Epidemiology and Disease Informatics, Bangalore 560 064, India, 560064

**Keywords:** E gene, envelope, KFD, KFDV, Kyasanur forest disease, Kyasanur forest disease virus, sequence analysis, vaccine

## Abstract

The present study was undertaken with the objective of performing comparative sequence analysis of the envelope (E) gene of Kyasanur forest disease virus (KFDV) vaccine strain P9605 with the circulating field strains. The study was taken up as the currently used KFDV vaccine strain, KFDV P9605, was isolated in the 1960s. For this study, we designed two sets of primers targeting the complete amplification of the E gene of KFDV. The sequencing was performed by the Sanger method, and the deduced sequence of the vaccine virus was deposited in GenBank with accession number PX067005. This sequence obtained for the vaccine virus was aligned and compared with sequences of GenBank‐deposited circulating field strains. We also performed comparative sequence analysis of the Kyasanur forest disease (KFD) vaccine seed virus having passaged twice in mouse brain with the vaccine seed virus passaged six times in mouse brain to investigate whether multiple passages in mouse brain will lead to genetic mutation in the immunologically important E gene. The phylogenetic analysis revealed seven amino acid mutations in the field strains at positions A123T, S158N, D178E, D239N, A313S, M429I, and G479A when compared with the vaccine strain. We did not find any mutations at the critical fusogenic segment (residues 98–113) in the E gene of currently circulating field strains compared to the vaccine seed virus. The study found no genetic variations in the E gene of the KFD virus passed two times and passed six times in mouse brain. We performed SWISS‐MODEL homology modeling, AlphaFold protein analysis, and Ramachandran plot analysis to study the E protein structures and stability. The observations made in this study suggest slow evolutionary drift and conserved structural stability of the envelope gene of KFDV ever since its emergence 7 decades ago; however, the functional implications of these amino acid substitutions need further studies on their roles in viral infectivity, transmission, and impact on immunity.

## 1. Introduction

Kyasanur forest disease (KFD) was first identified in 1957 in the Kyasanur forest region of Karnataka’s Shivamogga district, following widespread fatalities among two species of monkeys, viz., black‐faced langurs (*Presbytis entellus*) and red‐faced bonnet monkeys (*Macaca radiata*). The outbreak in monkeys coincided with severe febrile illness and deaths in humans, leading to the recognition of a new zoonotic disease, now commonly referred to as “monkey fever” [1, 2]. KFD is caused by Kyasanur forest disease virus (KFDV) belonging to the virus family *Flaviviridae* (1). The disease has been controlled primarily by using formalin‐inactivated tissue culture KFD vaccine produced using the KFD seed virus P9605 isolated in the 1960s. The disease was limited to the Western Ghats of Karnataka state, India, for about 7 decades; however, in the last decade, cases have been reported in all the states that share borders with Western Ghats forests [3]. It is now known that the infected hard tick, *Haemophysalis spinigera*, from the virus‐infected/dead monkeys, latches on to the humans, and the bite of the virus‐infected tick results in the transmission of the disease; other mammals like rodents, shrews, and cattle are speculated to be reservoirs of the virus [4–6].

The KFD virus has a positive‐sense, single‐stranded RNA genome of approximately 11 kb in size, encoding a single 3416 amino acid polyprotein, which is cleaved post‐translationally into three structural (capsid C, precursor membrane [prM] protein, and envelope E) and seven nonstructural (NS1, NS2a, NS2b, NS3, NS4a, NS4b, and NS5) proteins [6, 7]. The envelope glycoprotein E is central to the biology of KFDV. Like E proteins in other flaviviruses, it facilitates viral attachment to host cell receptors and entry through membrane fusion. Additionally, it triggers immune responses by stimulating the production of protective and neutralizing antibodies. Because of these properties, the E protein is a key antigen for developing vaccines and diagnostic tests [7, 8]. The E protein of flaviviruses is a beta‐class protein and consists of three domains. The central domain I (DI) connects the extended domain II (DII) with the globular domain III (DIII) and helps in receptor binding, as known in the case of other flaviviruses such as Dengue and Japanese encephalitis (JE), where the B‐cell epitopes occur in the domains DI and DII [9]. Understanding the molecular levels of the process of neutralization or escape is critical for successful development and improvement of vaccines. Hence, the study of the structural and functional aspects of the KFDV E‐protein is vital for the understanding of virus‐cell interactions as well as the biology of the virus. Taking these factors into consideration, the present study was undertaken with the objective of comparative sequence analysis of the envelope gene of the vaccine strain of KFD virus with field strains.

## 2. Materials and Methods

### 2.1. Procurement of KFD Virus

We procured the mouse brain suspension containing KFD seed virus of strain P9605 from the Indian Council of Medical Research‐National Institute of Virology (ICMR‐NIV), Pune. The seed virus provided had undergone six passages in the mouse brain.

### 2.2. Primer Designing

The full E gene spans 1488 base pairs (from nucleotide 846 to 2331), which was too long to amplify efficiently using a single primer pair. Hence, we designed two primer sets to ensure complete amplification of the E protein gene. The sequences used for designing primers were chosen based on diverse parameters, including year of collection, host species, and geographic origin, as detailed in Supporting file‐S1. The Sequence alignment was performed using the MUSCLE algorithm [10] in MEGA Version 12 [11]. Conserved regions flanking both ends of the envelope gene were identified and selected to facilitate the successful amplification of the full‐length E gene. To facilitate better understanding and analysis, the above selected region was divided into two segments, viz., E1 (from nucleotide 846 to 1560) and E2 (from nucleotide 1561 to 2331). Primer design was performed using the Geneious software. For the E1 gene, a conserved sequence located upstream of the envelope gene was selected as the starting point. In contrast, for the E2 gene, the sequence began just upstream of the reverse complement of the reverse primer (Supporting file‐S2). The designed primers were further evaluated using the IDT Primer Design tool to assess their GC content and to predict the likelihood of forming secondary structures such as hairpins, self‐dimers, and hetero‐dimers. This analysis confirmed that the selected primers exhibited high structural stability. The specificity of the primer sequences was validated *in silico* using the NCBI Primer‐BLAST tool [12] on the NCBI nonredundant nucleotide database, which showed 100% homology with the envelope (E) gene nucleotide sequences of KFD viruses deposited in GenBank, and the designed primers had 0% homology with unintended targets in the NCBI database.

### 2.3. Reverse Transcription–Polymerase Chain Reaction (RT‐PCR)

Extraction of viral RNA from the mouse brain suspension containing KFD seed virus was done as per the procedure described in the QIAamp Viral RNA extraction kit (Qiagen, USA) as previously described in [13]. The cDNA was synthesized from RNA extracted from the KFD seed virus using the cDNA synthesis master mix (TAKARA, Japan) with the reaction components as detailed in Supporting file‐S3 and the cycling conditions outlined in Supporting file‐S4.

RT‐PCR was performed to amplify the E gene using the primers designed in this study. To optimize the annealing temperatures for the newly designed primers in this study, a gradient PCR was performed. The PCR amplicons were visualized through agarose gel electrophoresis. Bluetongue virus cDNA was used as a negative control during the PCR reaction.

### 2.4. Sequence Analysis

The PCR products that yielded specific amplification of E1 and E2 genes were sequenced by the Sanger method [14] at M/s Sakala enterprises, Bengaluru, India. The resulting sequences were assessed for quality through chromatogram analysis. Sequences generated from the forward primers were aligned with the reverse‐complemented sequences of their respective reverse primers using the MUSCLE algorithm in MEGA Version 12.0.11, resulting in two finalized sequence sets. These were subsequently aligned to generate a complete envelope gene sequence. The finalized full‐length envelope gene sequence was submitted to NCBI‐GenBank.

Twenty full‐length envelope gene sequences of currently circulating KFDV field strains were retrieved from GenBank, representing diverse geographic origins where the disease has been endemic in the past decade (States of Karnataka, Maharashtra, Goa, and Kerala), different host species from which the virus has been isolated (Monkeys, Ticks, and Humans), and collection years spanning from 2015 to the present. These sequences were aligned with the sequence deduced for the KFD seed virus. Additionally, the envelope gene sequence from KFD vaccine seed virus P9605 with two passages in the mouse brain, submitted by Pragya Yadav under accession number OQ866117, was included to assess potential mutational changes across mouse brain passages. For phylogenetic analysis, the envelope gene sequence of Alkhurma hemorrhagic fever virus (accession number OR785376) was used as an outgroup. The list of all the sequences used for the phylogenetic analysis during this study is provided in Supporting file‐S5.

All selected sequences were aligned using the MUSCLE algorithm. MEGA Version 12.0.11 was employed for sequence alignment, model selection, and phylogenetic tree construction. The phylogenetic tree was generated using the Maximum Likelihood method based on the Tamura–Nei model [15].

The obtained nucleotide sequences were translated to amino acid sequences using MEGA Version 12.0.11 [11]. The complete amino acid sequence of the envelope (E) protein from the KFD seed virus was analyzed and compared with sequences from field strains. The mutation sites were identified using deduced sequences in MEGA Version 12.0.11.

We mapped the seven mutations to experimentally verified epitopes in the Immune Epitope Database (IEDB) [16]. Since no experimentally verified epitopes are available for KFDV in IEDB, we mapped the mutations to epitopes reported in related flaviviruses that share conserved envelope protein domains (e.g., TBEV, YFV, DENV).

The bioinformatics tool mCSM was employed [17, 18] to predict the potential impact of the seven amino acid substitutions on protein stability and antigenicity.

A three‐dimensional structural model of the KFDV E protein was generated using the SWISS‐MODEL tool (https://swissmodel.expasy.org/interactive#structure) [19] with the template 7z51.1.A. Global model quality estimate of the SWISS‐MODEL tool used was 0.85, which was a high score (range is 0–1), indicating strong reliability of the model based on the template. The QMEANDisCo global quality of the tool used was 0.78 ± 0.05, suggesting that the model had good agreement with expected structural features (values closer to 1 indicate higher quality). The functional domains and mutation sites were depicted separately using the EzMol tool (https://www.sbg.bio.ic.ac.uk/ezmol/).

We performed a direct comparison between AlphaFold [20] and SWISS‐MODEL predictions. The AlphaFold model (496 aa, average pLDDT 83.66) was aligned with the SWISS‐MODEL homology model (QMEANDisCo Global 0.78 ± 0.05, template sequence identity 81.05%) and was superimposed.

Additionally, to assess the structural integrity of the E protein, we performed Ramachandran plot analysis for the obtained amino acid sequences as per standard protocols [21].

## 3. Results

### 3.1. Primer Designing

The details of the primer sequences designed to amplify the full‐length sequence of the E gene, along with their genomic positions, annealing temperatures, and relevant properties, are detailed in Table 1.

**TABLE 1:** Properties of the designed primers during this study to amplify the full‐length envelope gene of KFDV.

E gene	Primer	Primer sequence (5′‐3′)	Annealing temperature standardized	Nucleotide length	Gene position	Product size (bp)
E1 (from 846 to 1560)	Forward primer 1	CTGGCGGTAGAAACAGGAGG	58°C	20	730 to 749	865
	Reverse primer 1	TCCGCTGTCTTGTCCAGTTC		20	1594 to 1575	

E2 (from 1561 to 2331)	Forward primer 2	CGGGTGACTAGTGGAGTGGA	60°C	20	1533 to 1552	979
	Reverse primer 2	CACCACATCGTAGCTCCATCC		21	2512 to 2491	

### 3.2. KFDV RNA Extraction, cDNA Synthesis, and RT‐PCR

**FIGURE 1:**
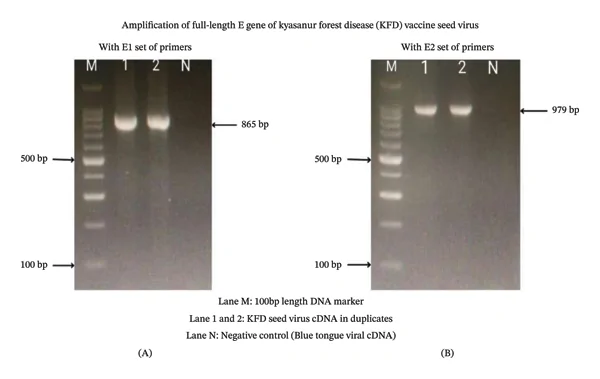
We designed the primers to amplify the full‐length E gene of the Kyasanur forest disease vaccine seed virus. The RT‐PCR performed on cDNA from the KFD seed virus using the primers for the E1 gene yielded a specific amplicon of 865 bp (Figure 1A), and using the primers for the E2 gene yielded a specific amplicon of 979 bp (Figure 1B).

A total volume of 50 μL of RNA of the KFD seed virus was extracted from the KFD seed stock as explained previously. A gradient PCR was performed to determine the optimal annealing temperatures for the newly designed primers. The results indicated that an annealing temperature of 58°C produced specific amplification for the primers targeting the E1 gene, while 60°C was optimal for the primers designed for the E2 gene (Supporting files—S6 and S7). The components of the master mix used for the PCR are listed in Supporting file‐S8. The RT‐PCR thermal cycling conditions included an initial denaturation at 94°C for 5 min, followed by 35 repeated cycles of denaturation at 94°C for 1 min, annealing (58°C for E1 primers and 60°C for E2 primers) for 1 min, and extension at 72°C for 1 min; a final extension was performed at 72°C for 5 min. The primers designed for the E1 gene produced a distinct amplicon of 865 bp, while those for the E2 gene generated a specific amplicon of 979 bp, as represented in Figure 1A, B, respectively.

### 3.3. Sequence Analysis

The PCR amplicons generated using the designed primers were submitted for sequencing. Upon receiving the resulting sequences, the sequences corresponding to the forward primers were aligned with the reverse complements of their respective reverse primers to facilitate accurate sequence assembly. This approach resulted in a unified contiguous sequence of the whole envelope (E) gene of the KFD seed virus as described earlier. The finalized full‐length E gene was submitted to NCBI GenBank, and sequences submitted were assigned the accession number PX067005.

**FIGURE 2:**
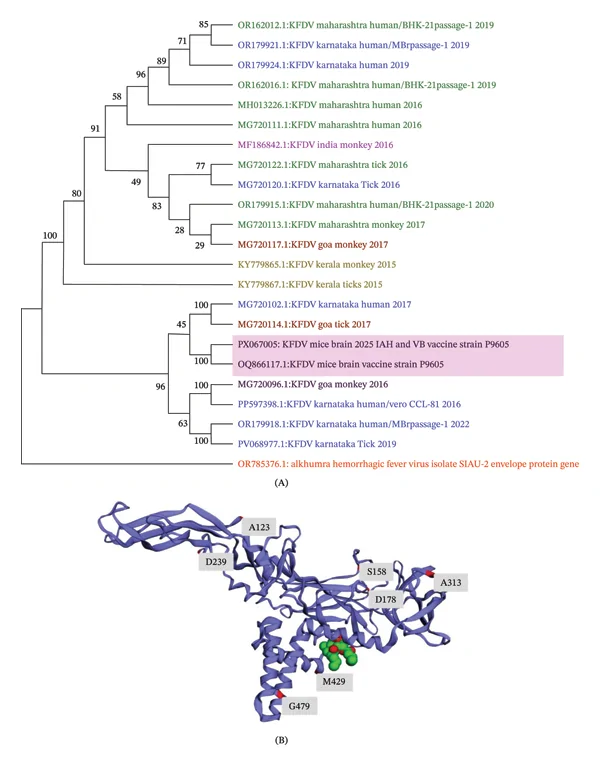
The finalized full‐length E gene was submitted to NCBI GenBank and received the accession number PX067005. The sequences obtained for the new KFD seed virus were aligned with those of recently circulating field strains, as well as with the previously reported passage‐derived sequence (GenBank accession no. OQ866117). The phylogenetic tree was constructed using the Maximum Likelihood method with a Bootstrap value of 1000 replicates. The resulting tree is presented in Figure 2A. A three‐dimensional structural model of the KFDV envelope protein was generated using SWISS‐MODEL software. This model enabled mapping of mutation sites observed in circulating field strains using the EzMol tool and is shown in Figure 2B. (A) Phylogenetic analysis of the full‐length Envelope gene sequences of Kyasanur forest disease virus. (B) 3D model of the envelope protein of the new KFD seed virus with mutations compared to field strains. The regions highlighted in red on the structure represent the locations of observed mutations.

The sequences obtained for the KFD seed virus in this study were aligned with those of recently circulating field strains, as well as with the KFD virus sequences deposited under GenBank accession number OQ866117 (with lower mouse brain passage submitted by Pragya Yadav), as described previously, and a phylogenetic tree was constructed using the Maximum Likelihood method (Figure 2A).

The phylogenetic analysis revealed that P9605 vaccine seed virus sequences (GenBank accession no. OQ866117—mouse brain passage 2) and PX067005‐mouse brain passage 6 remained completely identical and clustered within a single clade, suggesting that serial passaging in mouse brain up to six passages may not introduce mutations in the E gene. However, the limitation of this study was that we performed the genetic studies only on KFDV passaged two times and six times in mouse brain and couldn’t perform similar sequencing studies on KFDV passaged thrice, four times, and five times in mouse brain.

The vaccine virus clade was closely related to several field strains isolated from Karnataka, Goa, and Kerala, including human hosts. The short branch lengths and high bootstrap support suggest strong sequence conservation in the E gene spanning several decades and across distant regions. The field strains from Maharashtra and Goa had modest divergence but remained within the same major lineage as the vaccine strains. No distinct outgroup or highly divergent clade was observed, implying that the circulating strains have not undergone significant antigenic drift in the E protein gene.

The translated sequences of all these strains were compared; results showed single amino acid substitutions at seven sites on the E gene of circulating field KFDV strains: A123T, S158N, D178E, D239N, A313S, M429I, and G479A; a list of these mutations is given in Table 2.

**TABLE 2:** List of mutations in ectodomains between vaccine strain and currently circulating KFDV field strains.

Amino acid position	Vaccine strain residue	Field strain residue	States observed	Years	Accession numbers
123	A	T	Karnataka, Maharashtra, Goa	2016–2019	MG720102, OR179921, OR179924, MH013226, MG720111, OR162012, OR162016, MG720114
158	S	N	Kerala	2015	KY779867
178	D	E	Karnataka	2019, 2022	PV068977, OR179918
239	D	N	Karnataka, Maharashtra, Kerala, Goa	2015–2020	MG720120, OR179921, OR179924, MH013226, MG720111, MG720122, MG720113, OR162012, OR162016, OR179915, KY779865, KY779867, MG720117
313	A	S	Karnataka	2019, 2022	PV068977, OR179918
429	M	I	Karnataka, Goa	2016–2017	PP597398, MG720102, MG720096, MG720114
479	G	A	Karnataka, Goa	2017	MG720102, MG720114

Mapping of these seven mutations to experimentally verified epitopes of related flaviviruses sharing conserved envelope protein domains in IEDB showed that residues S158N and A313S overlap validated T‐cell epitopes, while residues A123T, D178E, D239N, M429I, and G479A fall within B‐cell epitopes recognized in antibody assays. The details of the epitope mapping are listed in Table 3.

**TABLE 3:** Mapping of the seven mutations observed in this study to experimentally verified epitopes in the immune epitope database (IEDB).

Mutation position	Overlapping epitope	IEDB ID	Source virus	Epitope type	Notes
123	GKGSIVACVKAACEA	229659	TBEV	B‐cell epitope	Mutation at epitope terminus
158	ANETHSGRKTASFTI	229604	TBEV	T‐cell epitope	Mutation directly inside validated epitope
178	ILTMGEYGDVSLLCR	229685	TBEV	B‐cell epitope	Mutation overlaps domain II epitope
239	LPWKHEGAQNWNNAE	229729	TBEV	B‐cell epitope	Mutation overlaps hinge epitope
313	LKMKGLTYTMCDKTK	229722	TBEV	T‐cell epitope	Mutation at epitope end
429	FLSSIGKAVHTVLGG	229640	TBEV	B‐cell epitope	Mutation inside domain III epitope
479	AWLGLNMRNPTMSMS	229615	TBEV	B‐cell epitope	Mutation overlaps stem epitope

The bioinformatic tool mCSM, employed to predict the potential impact of the seven amino acid substitutions on protein stability and antigenicity, predicted that the mutation at A313S is the strongest destabilization substitution with ΔΔ*G* of −1.056 and is likely significant for antigenicity. The details of the predictions for the seven amino acid mutations are listed in Table 4.

**TABLE 4:** Prediction of the potential impact of the amino acid substitutions on protein stability and antigenicity using the bioinformatic tool mCSM.

Position	Mutation	RSA	ΔΔG	Interpretation
123	A ⟶ T	96.9	−0.446	Slight destabilization, surface‐exposed. Could affect local epitope.
158	S ⟶ N	106.6	−0.08	Essentially neutral, highly exposed, but minimal stability change.
178	D ⟶ E	76.1	−0.221	Conservative change, mild destabilization.
239	D ⟶ N	67.4	−0.572	Moderate destabilization may affect the antigenic surface.
313	A ⟶ S	74.6	−1.056	Strongest destabilization, likely to alter local structure/epitope.
429	M ⟶ I	88.2	+0.142	Slight stabilization, conservative change.
479	G ⟶ A	69.3	−0.264	Mild destabilization, surface‐exposed.

A three‐dimensional structural model of the KFDV envelope protein was generated using previously described computational tools. This model enabled mapping of mutation sites observed in circulating field strains, as shown in Figure 2B.

**FIGURE 3:**
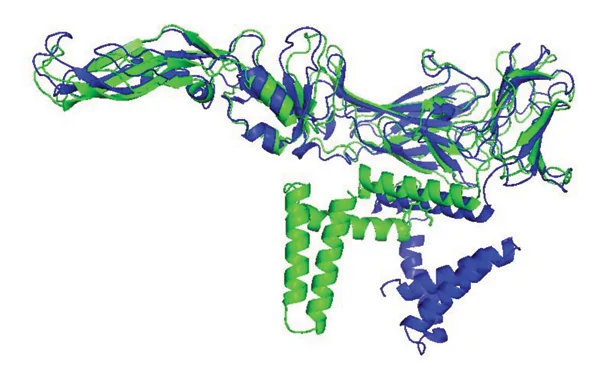
Superimposition of AlphaFold prediction (blue) and SWISS‐MODEL homology model (green) for the E protein of the vaccine strain of KFDV. Structural alignment yielded an RMSD of 2.6 Å, indicating good agreement in the conserved fold. AlphaFold provides full‐length coverage with high confidence (avg pLDDT 83.66), while SWISS‐MODEL shows good template‐based quality (QMEANDisCo 0.78 ± 0.05).

Superimposition of AlphaFold prediction (blue) and SWISS‐MODEL homology model (green) for the E protein of the vaccine strain of KFDV is shown in Figure 3. Structural alignment yielded a root mean square deviation of 2.6 Å, indicating good agreement in the conserved fold. AlphaFold provides full‐length coverage with high confidence (avg pLDDT 83.66), while SWISS‐MODEL shows good template‐based quality (QMEANDisCo 0.78 ± 0.05).

The Ramachandran plot analysis of the total 492 amino acid residues found that 97%–98% of residues belong to the favored region, only ∼2% in the allowed regions, and no amino acid residues in the disallowed region, indicating excellent quality with strong structural integrity in the KFD vaccine strain (Supporting file‐S9: A, B, C, D). The Ramachandran plot analysis of the envelope protein amino acid sequences in the circulating field isolates showed similar structural integrity as seen in the envelope protein of the vaccine virus.

## 4. Discussion

The glycoproteins of the virus envelope have critical functions in the virus lifecycle, including receptor binding, membrane fusion, and entry into the host cell. Therefore, these proteins are important determinants of host range, tissue tropism, virulence, and, most importantly, induction of protective immunity [22]. Flaviviruses are positive‐sense RNA viruses that are amongst the smallest viruses that contain a lipid bilayer membrane with inserted glycoproteins on their surfaces. Most of these viruses are transmitted to their vertebrate host by infected mosquitoes or ticks. Several of these viruses are significant human pathogens, including Yellow fever virus, JE virus, Dengue virus, and KFDV [23].

The KFDV RNA genome encodes a total of 14 proteins: seven structural and seven nonstructural. The structural proteins include the core capsid (C) with 351 nucleotides, the envelope protein (E) with 1488 nucleotides, and the prM protein with 492 nucleotides. The nonstructural proteins are NS1 (1059 nucleotides), NS2A and NS2B combined (1083 nucleotides), NS3 (1863 nucleotides), NS4A and NS4B combined (1203 nucleotides), and NS5 (2705 nucleotides). The gene sequence follows the order: 5′‐C‐prM(M)‐E‐NS1‐NS2A‐NS2B‐NS3‐NS4A‐NS4B‐NS5–3′. Based on the complete genome, KFDV encodes 3416 amino acids in total, with the envelope protein alone contributing 496 amino acids [24]. Immunologically, the E protein is highly significant, as it contains multiple epitopes that are recognized by both B and T cells. These B‐cell epitopes can trigger the production of neutralizing antibodies, while the protein also promotes T‐cell responses [25].

Considering these immunological factors and also the fact that the KFD vaccine seed virus P9605 is more than seven decades old, we amplified the full‐length envelope gene of the KFD vaccine seed virus and performed its comparative sequence analysis in relation to recently circulating field strains of KFDV. Upon comparing the amino acid sequence of the vaccine virus with 20 currently circulating field strains, we identified single nucleotide polymorphisms in some strains, resulting in amino acid substitutions at seven positions: A123T, S158N, D178E, D239N, A313S, M429I, and G479A. The presence of these mutations even in the native group of KFD virus strains from Karnataka indicates that these mutations are unlikely to be linked to the virus adapting to the local/new environment. Instead, these genetic changes may have aided the spread of the virus to new regions by offering some benefit in its interaction with vectors or hosts. It is also possible that these mutations are simply the result of random genetic variations.

The polypeptide chain of the E protein folds into three distinct domains: a central β‐barrel structure (DI), an extended region responsible for dimerization (DII), and a C‐terminal domain resembling an immunoglobulin fold (DIII). Out of the three domains, DII is immunologically important, as it contains multiple epitopes that are recognized by both B and T cells. The cd loop at the tip of DII contains a sequence (residues 98–113) that is almost fully conserved in all flaviviruses and is important for the fusogenic activity of the virus [22, 23]. Consequently, any alterations in tissue tropism or disease severity of the KFDV are likely to involve mutations within this critical segment of the E glycoprotein [14]. The detailed comparative sequence analysis of the KFD vaccine seed virus and the circulating field strains evaluated in this study (from 2015 onwards) showed homologous sequences and did not find any mutations at this critical fusogenic site on the E gene (98–113 amino acid residues).

The SWISS‐MODEL homology modeling, AlphaFold protein analysis, and Ramachandran plot analysis employed in this study indicate similarity in the structural stability of the E protein between circulating field isolates and the vaccine virus. These findings augment the fact that tick‐borne flaviviruses exhibit relatively slow evolutionary rates compared to their mosquito‐borne counterparts, which often undergo rapid genetic changes and are dispersed over long distances through migratory birds, humans, animals, or even mosquito eggs [26]. Taken together, these findings possibly imply that the KFD vaccine produced using seed virus strain P9605 has helped significantly reduce disease morbidity and mortality in KFD endemic areas, in concurrence with several previous reports [3, 27–29].

The genetic similarity of the E gene of the KFD virus after the second passage in the mouse brain with the KFD virus having six passages in the mouse brain possibly indicates that sequential passaging in the mouse brain up to six passages may not induce genetic mutation in the immunologically significant envelope gene, obliterating any apprehension that multiple passages of the seed virus in mouse brain may reduce the vaccine efficacy. Further, we also showed for the first time that the envelope gene of KFDV remained conserved and stable at the critical fusogenic site even after 7 decades of emergence of KFD virus, demonstrating the genomic resilience in tick‐borne flaviviruses in general and KFDV in particular.

The findings in our study on the amino acid substitutions were in concurrence with Yadav and coworkers [9] who also showed a minor substitution at position A123T in the circulating KFD viruses, and with Mehla and coworkers [8] who revealed minimal genetic variations of 1.2% at the nucleotide level and 0.5% at the amino acid level in the currently circulating KFD viruses, which did not significantly impact the antigenic properties or three‐dimensional configuration of the KFD virus.

A recent work [30] on multiple sequence alignment analysis for *in-silico* design of an envelope‐based multi‐epitope vaccine candidate against KFDV has shown that, except for a few amino acids, the entire KFDV envelope protein sequences have remained conserved in the circulating viruses, which is in accordance with the findings in this study. A comprehensive computational analysis of 73 complete genome sequences of KFDV between 1957 and 2022 has shown two distinct lineages of KFDV [31]. Lineage 1 includes KFDV isolate samples from Karnataka state during 1957–1972, and lineage 2 constituted the isolates sampled from Karnataka and Goa states between 2006 and 2022. The authors of this study have reported a genome‐wide nucleotide substitution rate of 4.31 × 0^−4^ substitutions per site per year through computational analysis [31]. However, the Ramachandran plot analysis of the multi‐epitope envelope protein‐based KFD vaccine candidate generated using this computational tool had 98.4% of amino acids in the favored region, which is in concurrence with the Ramachandran plot data of the amino acid sequences of the vaccine strain and circulating KFDV obtained in this study.

### 4.1. Limitations of the Study

The study involved only the immunologically important E gene/protein and did not take into consideration the other genes/proteins of the KFD virus, which could play important roles in immunity. We compared the sequences of KFD virus passaged twice and passaged six times in mouse brain, and we did not compare the sequences of intermediate mouse brain passages (three, four, and five). We used only bioinformatic tools to predict the impact of the seven mutations observed in the circulating KFD strains compared to the vaccine strain, and the study did not perform functional tests to validate the impact of the observed mutations.

## 5. Conclusions

The study involved a comparative sequence analysis of the envelope (E) gene of KFDV vaccine strain P9605 with the circulating field strains, which found single amino acid substitutions at seven locations on the E gene of circulating KFD viruses compared to the vaccine strain KFDV P9605. The study did not find any mutations at the critical fusogenic site (98–113 amino acid residues) on the E gene, demonstrating the genomic resilience in tick‐borne flaviviruses. This study also showed E gene sequence identity between KFDV passaged twice and six times in mouse brain. The SWISS‐MODEL homology modeling, AlphaFold protein analysis, and Ramachandran plot analysis employed in this study indicate similarity in the structural stability of the E protein between circulating field isolates and the vaccine virus. The immunological significance of the mutations observed in the circulating strains requires further investigation, since the IEDB mapping and bioinformatic tools used to predict the impact of these mutations have depicted that a few of these mutations can affect the E protein stability and antigenicity.

## Funding

The study was partly funded by Vision Group on Science and Technology (VGST), Department of Science and Technology, Govt. of Karnataka. CISEE Project GRD No. 1015.

## Conflicts of Interest

The authors declare no conflicts of interest.

## Supporting Information

Additional supporting information can be found online in the Supporting Information section.

## Supporting information


**Supporting Information** Legends for Supporting Files. S1: List of the sequences used for primer design. S2: Locations of the designed primer binding sites of E1 and E2 gene segments on the envelope gene. S3: Components of the master mix used for cDNA synthesis of KFD virus. S4: The cDNA synthesis temperature‐cycling conditions. S5: List of the KFD virus field strains considered for phylogenetic analysis in the study. S6: The RT‐PCR cycle conditions adopted for the amplification of the E1 gene. S7: The RT‐PCR cycle conditions adopted for the amplification of the E2 gene. S8: Components of RT‐PCR master mix used for amplification of KFD virus. S9: Ramachandran plot analysis of the amino acid sequences of the KFD vaccine virus.

## Data Availability

The original contributions presented in the study are included in the article. Further inquiries can be directed to the corresponding author.
