# Supplementary material for: Comparative Sequence Analysis of the Envelope Gene of Kyasanur Forest Disease Virus Vaccine Strain With Currently Circulating Field Strains
Source: Adv Virol. 2026 Sep 18;2026:6915402. doi: 10.1155/av/6915402 (PMC13589198; doi:10.1155/av/6915402)
Supplement: Supplementary file 1 [file AV-2026-6915402-s001.docx]

**Supplementary files**

**S1: List of the sequences used for primer designing**

| **Sl. No.** | **Accession no.** | **Year of  collection** | **Host** | **Isolate** | **Place** |
| --- | --- | --- | --- | --- | --- |
| 1. | JF416958.1 | 1957 | *Homo sapiens* | P9605 | India |
| 2. | MG720079 | 2012 | *Homo sapiens* | 121863 | Shivamogga district, Karnataka |
| 3. | KY779855.1 | 2012 | *Homo sapiens* | NIV12869 | Shivamogga district, Karnataka |
| 4. | MG720081.1 | 2013 | *Homo sapiens* | 131152 | Shivamogga district, Karnataka |
| 5. | KY779866.1 | 2013 | *Homo sapiens* | NIV135724 | Kerala |
| 6. | OR179910.1 | 2014 | *Homo sapiens* | NIV142856_KA_P1_10842 | Shivamogga district, Karnataka |
| 7. | KY779864.1 | 2015 | *Macaca radiata* | NIVAN152326 | Kerala |
| 8. | KY779867.1 | 2015 | *Haemophysalis* | MCL-15-T-338 | Kerala |
| 9. | [MG720118.1](https://www.ncbi.nlm.nih.gov/nucleotide/MG720118.1?report=genbank&log$=nucltop&blast_rank=6&RID=U1KDPF2V013) | 2016 | *Homo sapiens* | MCL_16_T_343 | Sindhudurg district, Maharashtra |
| 10. | PP597397.1 | 2016 | *Homo sapiens* | NIV_164187_plaque_1.1 | Belagavi, Karnataka |
| 11. | PP763477.1 | 2016 | *Homo sapiens* | NIV_164187_Plaque-1.1 | Belagavi, Karnataka |
| 12. | [OR162008.1](https://www.ncbi.nlm.nih.gov/nucleotide/OR162008.1?report=genbank&log$=nucltop&blast_rank=5&RID=U1KDPF2V013) | 2019 | *Homo sapiens* | MCL-19-H-552_MH_P1_11468 | Sindhudurg district, Maharashtra |
| 13. | OR179915.1 | 2020 | *Homo sapiens* | MCL-20-H-641 _MH_P1_11195 | Sindhudurg district, Maharashtra |
| 14. | OP037814.1 | 2022 | *Homo sapiens* | P9605 | India |
| 15. | OQ866116.1 | 2023 | *Homo sapiens* | P-9605-M-92-M-20543_MBR_P1_11524 | India |

**S2:	Locations of the designed primer binding sites of E1 and E2 gene segments on the envelope gene**

**S3: Components of master mix used for cDNA synthesis** **KFD virus**

| **Component** | **Volume per reaction (µl)** |
| --- | --- |
| 5X buffer | 4.0 |
| Hexamer | 1.0 |
| Enzyme | 1.0 |
| dNTP | 1.0 |
| RNase inhibitor | 0.5 |
| RNA | 3.0 |
| NFW | 9.5 |
| **Total** | **20** |

**S4: The cDNA synthesis temperature-cycling conditions**

| Random hexamers binding | 30 °C for 10 min |
| --- | --- |
| cDNA synthesis | 42 °C for 60 min |
| Inactivating the enzyme | 95 °C for 5 min |
| Cooling | 4 °C for 5 min |

**S5: List of the KFD virus field strains considered for phylogenetic analysis in the study**

| **Sl. No.** | **Accession no.** | **Year of collection** | **Host** | **Place** |
| --- | --- | --- | --- | --- |
| 1. | KY779865 | 2015 | Monkey | Kerala |
| 2. | MF186842 | 2016 | Monkey | India |
| 3. | MG720111 | 2016 | Human | Maharashtra |
| 4. | MG720120 | 2016 | Tick | Shivamogga, Karnataka |
| 5. | MG720122 | 2016 | Tick | Maharashtra |
| 6. | MH013226 | 2016 | Human | Maharashtra |
| 7. | PP597398 | 2016 | Human | Belagavi, Karnataka |
| 8. | MG720096 | 2016 | Monkey | Goa |
| 9. | MG720102 | 2017 | Human | Shivamogga, Karnataka |
| 10. | MG720113 | 2017 | Monkey | Maharashtra |
| 11. | MG720114 | 2017 | Tick | Goa |
| 12. | MG720117 | 2017 | Monkey | Goa |
| 13. | KY779867 | 2017 | Tick | Kerala |
| 14. | OR179924 | 2019 | Human | Shivamogga, Karnataka |
| 15. | OR162016 | 2019 | Human | Maharashtra |
| 16. | OR179921 | 2019 | Human | Shivamogga, Karnataka |
| 17. | OR162012 | 2019 | Human | Maharashtra |
| 18. | PV068977 | 2019 | Tick | Sagara Taluk, Karnataka |
| 19. | OR179915 | 2020 | Human | Maharashtra |
| 20. | OR179918 | 2022 | Human | Shivamogga, Karnataka |

**S6: The RT-PCR cycle conditions adopted for the amplification of E1 gene**

| Initial denaturation | 94 °C for 5 min |
| --- | --- |
| Final denaturation | 94 °C for 1 min |
| Annealing (35 cycles) | 58 °C for 1 min |
| Initial extension | 72 °C for 1 min |
| Final extension | 72 °C for 5 min |
| Cooling | 4 °C for 5 min |

**S7: The RT-PCR cycle conditions adopted for the amplification of E2 gene**

| Initial denaturation | 94 °C for 5 min |
| --- | --- |
| Final denaturation | 94 °C for 1 min |
| Annealing (35 cycles) | 60 °C for 1 min |
| Initial extension | 72 °C for 1 min |
| Final extension | 72 °C for 5 min |
| Cooling | 4 °C for 5 min |

**S8: Components of RT-PCR master mix used for amplification of KFD virus**

| **Component** | **Volume per reaction (µl)** |
| --- | --- |
| Reaction mix | 12.5 |
| Forward primer | 1.0 |
| Reverse primer | 1.0 |
| cDNA | 1.0 |
| Enzyme mix | 1.0 |
| NFW | 8.5 |
| **Total** | **25** |

**S9: Ramachandran plot analysis of the amino acid sequences of the KFD vaccine virus**

**A.** **2D Ramachandran plot of six distinct categories:**

(a) general case (Ala and remaining 15 amino acids),

(b) Gly, (c) Val/Ile, (d) pre-Pro, (e) trans-Pro & (f) cis-Pro.

Here, cyan blue and red dots represent torsion angles of favoured, allowed and disallowed regions

respectively.

**B.** **Standard 2D Ramachandran Plot**
 Here, cyan, blue and red (dots/triangles) represent torsion angles of favoured, allowed and disallowed

regions respectively; dot represents residues other than glycine and triangles represents glycine.

**C**. **3D Ramachandran plot of six distinct categories**:

(a) general case (Ala and remaining 15 amino acids),

(b) Gly, (c) Val/Ile, (d) pre-Pro, (e) trans-Pro & (f) cis-Pro.
 Here, bars represent frequency of torsion angles.

**D.** **Standard 2D Ramachandran Plot**
 Here, cyan, blue and red (dots/triangles) represent torsion angles of favoured, allowed and disallowed

 regions respectively; dot represents residues other than glycine and triangles represents glycine.

**Summary of Results of the Ramachandran plot analysis**

Total residues analysed: 492

**Standard statistics:**

Favored: 484 residues (98.37%)

Allowed: 8 residues (1.63%)

Disallowed: 0 residues (0%)

**Six-category statistics**:

Favored: 482 residues (97.97%)

Allowed: 10 residues (2.03%)

Disallowed: 0 residues (0%)

**Seven-category statistics**:

Favored: 482 residues (97.97%)

Allowed: 10 residues (2.03%)

Disallowed: 0 residues (0%)

**Interpretation**

- Excellent quality:
- More than 97–98% of residues are in favoured regions.
- Only ~2% in allowed regions, which is acceptable.
- No disallowed residues at all — this is a very strong indicator of structural validity.
